# Magnetic CuFe_2_O_4_ Spinel–Polypyrrole Pseudocapacitive Composites for Energy Storage

**DOI:** 10.3390/ma17215249

**Published:** 2024-10-28

**Authors:** Mahmoud Awad, Igor Zhitomirsky

**Affiliations:** Department of Materials Science and Engineering, McMaster University, Hamilton, ON L8S 4L7, Canada; awadm11@mcmaster.ca

**Keywords:** spinel, copper ferrite, polypyrrole, composite, supercapacitor, hydrothermal synthesis, ferrimagnetism

## Abstract

This investigation focused on the fabrication of ceramic ferrimagnetic CuFe_2_O_4_–conductive polypyrrole (PPy) composites for energy storage. CuFe_2_O_4_ with a crystal size of 20–30 nm and saturation magnetization of 31.4 emu g^−1^ was prepared by hydrothermal synthesis, and PPy was prepared by chemical polymerization. High-active-mass composite electrodes were fabricated for energy storage in supercapacitors for operation in a sodium sulfate electrolyte. The addition of PPy to CuFe_2_O_4_ resulted in a decrease in charge transfer resistance and an increase in capacitance in the range from 1.20 F cm^−2^ (31 F g^−1^) to 4.52 F cm^−2^ (117.4 F g^−1^) at a 1 mV s^−1^ sweep rate and from 1.17 F cm^−2^ (29.9 F g^−1^) to 4.60 F cm^−2^ (120.1 F g^−1^) at a 3 mA cm^−2^ current density. The composites showed higher capacitance than other magnetic ceramic composites of the same mass containing PPy in the same potential range and exhibited improved cyclic stability. The magnetic behavior of the composites was influenced by the magnetic properties of ferrimagnetic CuFe_2_O_4_ and paramagnetic PPy. The composites showed a valuable combination of capacitive and magnetic properties and enriched materials science of magnetic supercapacitors for novel applications based on magnetoelectric and magnetocapacitive properties.

## 1. Introduction

Ceramic–conductive polymer composites have attracted rising attention due to their advanced multifunctional properties and interesting interface effects, which result in enhanced magnetoresistance, conductivity, magnetization, charge storage, and other properties [1,2,3,4]. Moreover, the addition of conductive polymers to ceramics improves the processability of ceramic–polymer nanocomposites [4]. Conductive polymers show valuable capacitive properties for energy storage applications [5,6,7,8,9,10]. (LaSr)MnO_3_ (LSMO)–polyaniline (PANI) composites showed enhanced conductivity and magnetoresistance in [1]. It was demonstrated that the synergistic effects in the composites resulted from the influence of the PANI coating on the LSMO particles on the electric transport and magnetoelectric phenomena in the magnetic fields. A magnetoresistance increase of ~73% was reported in the composites containing LSMO particles coated with PANI [11]. Polypyrrole (PPy) coatings on LSMO particles were developed in [3], and such coatings influenced the charge transfer, double-exchange interactions, and magnetization. Of particular interest was the significant enhancement in magnetization from 49.4 to 102.0 emu g^−1^ of the core–shell particles due to the decreased spin disorder in the LSMO core [3]. The magnetization increase resulted from the transfer of charge and spin from the conductive polymer layer to the LSMO. An enhancement of saturation magnetization was also reported for PPy-coated Fe_3_O_4_ particles and attributed to charge transfer, which increased the surface magnetic moment of the particles [12]. An investigation of the charge storage properties of LSMO-PPy composites showed a considerable increase in electrochemical capacitance compared with pure PPy [13]. Magnetoelectric composites have been developed for diverse applications in solar cells, memory devices, and sensors [4]. CoFe_2_O_4_-PPy composites showed advanced microwave absorption properties in [14]. ZnO-PEDOT composites exhibited promising properties for applications in electrochromic displays in [15].

Efficient CuO-PPy anodes were developed for energy storage in lithium-ion batteries in [16,17,18,19]. The composites offered the advantages of increased conductivity and capacity. Moreover, the detrimental volume changes related to charging and discharging were reduced. CuO-PPy composites showed promising performance for applications in supercapacitors with enhanced capacitance [20,21,22]. The investigations in [23] revealed a synergy of the charge storage properties of CuO and PPy, which led to the high capacitance of the composites. Hexagonal ferrite [24] and spinel [25] ferrimagnetic ceramics were combined with PPy for the fabrication of advanced supercapacitor composite anodes. In addition to high capacitance, such composites exhibited valuable magnetic properties.

The progress achieved in the development of ceramic–conductive polymer composites indicates that further advances in this area can result in the development of novel advanced composites containing various functional ceramic materials. Composites exhibit valuable functional properties, such as advanced mechanical properties, photovoltaic properties, high conductivity, and advanced mechanical and electrical properties, which have been utilized for novel applications [26,27,28,29,30,31,32]. CuFe_2_O_4_ is a promising multifunctional material for the development of ceramic–polymer composites. CuFe_2_O_4_ is a spinel ferrimagnetic compound [33,34] that exhibits high magnetization due to superexchange interactions between Cu^2+^ and Fe^3+^ ions. It is an advanced material for biomedicine [35], catalytic applications [36], water purification [37], photovoltaic [38] devices, and energy storage applications in supercapacitors [39,40,41,42,43]. Therefore, the development of CuFe_2_O_4_–conductive polymer composites can result in materials with advanced functionality.

This investigation was motivated by the interesting multifunctional properties of ceramic–PPy composites. Moreover, the research motivation was fueled by the multifunctional properties of CuFe_2_O_4_ and recent advances in the development of multifunctional materials [44], combining high magnetization and remarkable pseudocapacitive properties. Such materials show novel magnetoelectric effects, which are currently under consideration for the fabrication of novel multifunctional devices. Of particular interest are the results regarding capacitance increases and charge transfer resistance decreases in a magnetic field, electric potential-controlled magnetization reversal, electric potential-controlled shift of the Curie point, periodic variations in magnetization by periodic galvanostatic charge–discharge, electric field control of saturation magnetization, and other magnetocapacitive and magnetoelectric effects [44]. Different mechanisms that provide a direct link between magnetization and redox reactions were described in [44]. As pointed out above, the combination of pseudocapacitive conductive polymers and ceramic magnetic particles generates new effects related to spin transfer from the paramagnetic polymers to the magnetically ordered ceramic materials, which results in enhanced magnetization.

The objective of this work was the fabrication and testing of new CuFe_2_O_4_-PPy composites. In contrast with other studies on magnetic complex oxide ceramic–PPy composites [24,25], which involved high-energy ball milling for the particle size reduction of the ceramic phase, the fabrication of CuFe_2_O_4_ nanoparticles was performed by hydrothermal synthesis. The composite materials combined high magnetic and pseudocapacitive properties due to the synergy of the contributions of CuFe_2_O_4_ and PPy. It was found that the magnetic properties of the composites were influenced by the weakly paramagnetic PPy. The combination of CuFe_2_O_4_ and PPy resulted in improved capacitance and cyclic stability.

## 2. Materials and Methods

Iron (III) chloride hexahydrate (FeCl_3_·6H_2_O), copper (II) chloride dihydrate (CuCl_2_·2H_2_O), trisodium citrate dihydrate (C_6_H_9_Na_3_O_9_) (TSCD), NaOH, polyvinyl butyral (PVB), pyrrole (Py), ammonium persulfate (APS), and 4,5-dihydroxy-1,3-benzenedisulfonic acid disodium salt monohydrate (Tiron) were supplied by Aldrich. Multiwalled carbon nanotubes (MWCNTs) (with outer and inner diameters of 12 and 5 nm, respectively, and an average length of 1.5 µm) were provided by Bayer, Germany. Ni foams (with a thickness of 1.5 mm and 95% porosity) were supplied by Vale, Ontario, Canada.

CuFe_2_O_4_ (CFO) and PPy were used for the fabrication of composite materials. CFO was selected due to its high magnetization and promising pseudocapacitive properties. PPy is an advanced pseudocapacitive material that offers the benefit of high conductivity.

The synthesis of CFO nanoparticles was performed with 7 mM of FeCl_3_ and 3.3 mM of CuCl_2_ as precursors. Both were dissolved in 50 mL of DI water. Then, TSCD was added as a chelating agent to the solution at 15% according to the weight of the precursors. The solution was stirred until completely dissolved. An aqueous solution of NaOH was used to adjust the pH to 12. The hydrothermal process was conducted for 8 h at 180 °C. The product was washed with DI water and ethanol several times to remove the unwanted by-products and dried for 12h at 55 °C.

The synthesis of PPy was achieved with a 0.1 M Py reagent added to DI water cooled with an ice bath to 4 °C. Tiron was added as a dopant to the solution with a Py:Tiron molar ratio of 10:1. Then, 0.1 M APS was added dropwise as an oxidizing agent. The mixture was kept under stirring for 2 h to complete the chemical reaction. Subsequently, the Tiron-doped PPy was washed with DI water to remove excess materials and dried until a constant mass was reached.

The synthesis methods used in this investigation facilitated the fabrication of stable slurries of PPyand CFO. The sedimentation tests showed the good colloidal stability of the slurries in ethanol, which facilitated improved the mixing of the individual components. Moreover, the use of Tiron as a dopant for PPy was beneficial for the improved mixing of PPy and CFO. It is known that catecholate-type materials, such as Tiron, provide strong chelating bonding to metal atoms on the surface of inorganic nanoparticles [45]. The slurries in ethanol for the impregnation of Ni foam current collectors contained the materials in a ratio of (CFO + PPY):MWCNT:PVB = 80:20:3. CFO100 samples were prepared without PPy as a control sample. Different composite materials were prepared by variations in the PPy and CFO contents in their composition to analyze the influence of the electrode composition on their properties. The preparation of electrodes with the same active mass loading and same thickness was important for the comparison of their properties. However, the limitations of this preparation method resulted from the low density of PPy. As a result, the maximum content of the PPy phase in the electrodes of the same mass and thickness was 70%. The samples CFO90, CFO70, CFO50, and CFO30 contained CFO and PPy. The CFO:PPy mass ratios were 90:10, 70:30, 50:50, and 30:70 for CFO90, CFO70, CFO50, and CFO30, respectively. The mass of the impregnated materials after drying was 40 mg cm^−2^. To increase physical contact, the impregnated foams were pressed to 37% of their original thickness. All electrodes had an area of 1 cm^2^.

Transmission Electron Microscopy (TEM) analysis was conducted using a Talos L120C system, while X-ray diffraction (XRD) patterns were recorded with a Bruker D8 Advance diffractometer (Cu-Kα radiation). Scanning Electron Microscopy (SEM) imaging was carried out with a JSM 6610LV (JEOL, Tokyo, Japan). Electrochemical investigations, including cyclic voltammetry (CV), electrochemical impedance spectroscopy (EIS), and galvanostatic charge–discharge (GCD) studies, were performed using a VMP 300 potentiostat (BioLogic, Seyssinet-Pariset, France). The magnetic data were obtained with MPMS SQUID magnetometer (Quantum Design, San Diego, CA, USA). The simulations of the EIS data were performed using the ZSimpWin software v3.6 (AMETEK, Oak Ridge, TN, USA). All electrochemical tests of the electrodes were conducted in a three-electrode setup using a 0.5 M Na_2_SO_4_ aqueous electrolyte. A saturated calomel electrode (SCE) served as the reference electrode, while a platinum gauze was used as the counter electrode. Equations (1) and (2) were used to calculate the integral capacitances (C_S_ and C_m_) by normalizing the total capacitance (C) by the electrode area and mass, respectively [46]:

(1) $$C=\frac{\Delta Q}{\Delta U}=\frac{|\int_{0}^{t(U_{max})}Idt|+|\int_{t(U_{max})}^{0}Idt|}{2U_{max}}$$

where I is the current, ΔQ is the charge, and ΔU is the potential range, and from the chronopotentiometry data:

C = IΔt/ΔU(2)

The complex capacitance C*(ω) = C′(ω) − iC″(ω) was derived from the EIS data at various frequencies (ω) of complex impedance, where Z*(ω) = Z′(ω) + i Z″(ω) [47]:

(3) $$C^{\prime }\left(\omega \right)=\frac{-Z^{\prime \prime }(\omega )}{\omega {\left|Z(\omega )\right|}^{2}}$$

(4) $$C^{\prime \prime }\left(\omega \right)=\frac{Z^{\prime }(\omega )}{\omega {\left|Z(\omega )\right|}^{2}}$$

## 3. Results and Discussion

**Figure 1:**
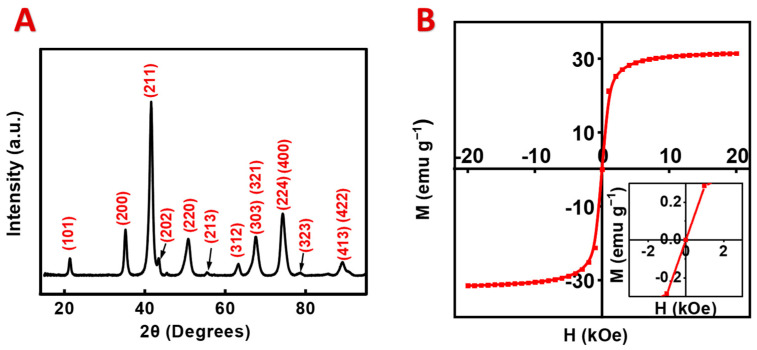
(**A**) X-ray X-ray diffraction pattern of CuFe_2_O_4_, prepared by hydrothermal synthesis. Miller indices of planes correspond to JCPDS file 00-034-0425. (**B**) Magnetization (M) plotted against magnetic field (H) for CuFe_2_O_4_ (inset shows low-field range).

The XRD diffraction pattern of the as-prepared material (Figure 1A) confirmed the formation of a pure spinel phase of CuFe_2_O_4_. The diffraction angles and peak intensities corresponded to JCPDS file 00-034-0425. The relatively wide peaks indicated a small particle size. The magnetic measurements (Figure 1B) showed a saturation magnetization of 31.4 emu g^−1^, which was higher than the saturation magnetization of CuFe_2_O_4_ prepared by some other methods [48,49]. It is known that the magnetization of CuFe_2_O_4_ depends on the occupancy of the tetrahedral and octahedral positions of the spinel crystallographic unit cells by the Cu^2+^ and Fe^3+^ ions. The literature data for this material show that saturation magnetization can be in the range of 1–2.3 µ_B_ per molecule [34]. The saturation magnetization of magnetic materials usually decreases with decreasing nanoparticle size [50]. The analysis of magnetization versus magnetic field dependence in low fields (Figure 1B, inset) did not show hysteresis. The zero net magnetic moment in the absence of a magnetic field indicated a superparamagnetic behavior, which can result from a small crystal size.

**Figure 2:**
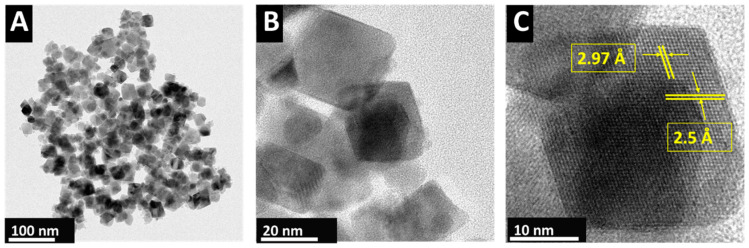
(**A**–**C**) TEM images of CuFe_2_O_4_ particles, prepared through hydrothermal synthesis. Spacings of 2.5 Å and 2.97 Å in the high-resolution image (**C**) correspond to d-spacings of crystallographic planes (211) and (112), respectively.

The TEM studies (Figure 2) showed that the hydrothermal synthesis resulted in the formation of CuFe_2_O_4_ nanoparticles with a size of 20–30 nm. The TEM images showed well-defined crystal faces. The high-resolution TEM image demonstrated the crystallinity of the obtained material. The small size of the CuFe_2_O_4_ particles was beneficial for the fabrication of composites.

**Figure 3:**
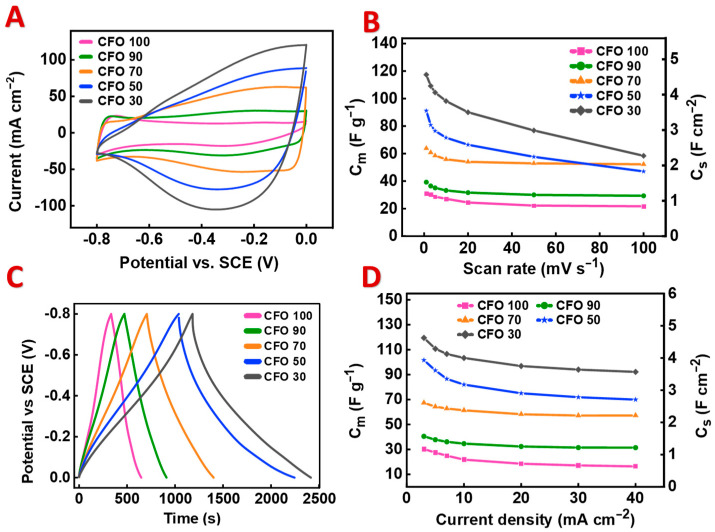
(**A**) CV data at 20 mV s^−1^, (**B**) capacitances at different scan rates, (**C**) GCD data at 3 mA cm^−2^, and (**D**) capacitances at different current densities for different composites.

The composite materials were tested for applications in supercapacitor electrodes. Figure 3 compares the electrochemical testing results for the different composites, and the results are summarized in Table 1. It is obvious that the CV areas increased with the increasing PPy content in the composites (Figure 3A), which indicated a capacitance increase (Figure 3B). The CVs did not show redox peaks, which are typical for battery-type behavior [51,52]. The charge storage mechanism of CuFe_2_O_4_ is related to the reduction in Fe^3+^ and Cu^2+^ ions [40]. The charge and discharge times also increased with the increasing PPy content (Figure 3C) due to the capacitance increase. The electrodes showed good capacitance retention with the increasing sweep rate and current density (Figure 3 and Table 1). CFO50 and CFO30 showed C_S_ values at 1 mV s^−1^ of 3.60 and 4.52 F cm^−2^, respectively, which were the highest capacitances of the composites studied in this investigation. The Cs values at 3 mA cm^−2^ of 3.90 and 4.60 F cm^−2^ were achieved for CFO50 and CFO30, respectively. As pointed out above, the fabrication of electrodes with the same thickness and same active mass loading presented difficulties for higher PPy content due to the lower density of PPy compared with the density of CuFe_2_O_4_.

**Figure 4:**
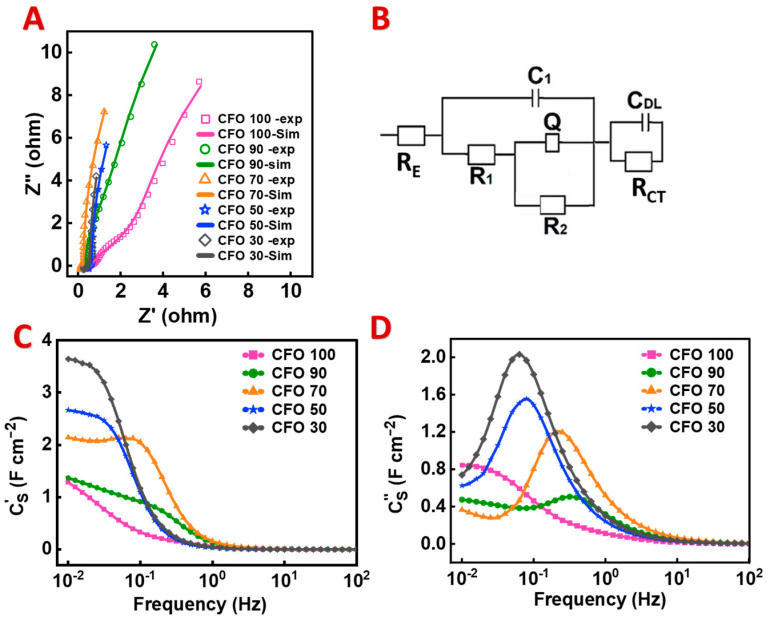
(**A**) Nyquist plots, (**B**) equivalent circuit model of EIS data, and (**C**,**D**) components of AC capacitance versus frequency.

Figure 4 shows the EIS data for the different composites. The Nyquist plot (Figure 4A) shows the impedance measurement data together with the simulation results obtained using the equivalent circuit presented in Figure 4B. The impedance decreased with the increasing PPy content. The real part of the impedance decreased due to the increased conductivity. The reduction in the imaginary part resulted from the increased capacitance. The equivalent circuit used for the simulation was developed for high-active-mass loading bulk electrodes [53]. It is known that porous electrodes can be described by transmission lines containing several RC elements [54,55,56,57]. The circuit presented in Figure 4B contains two RC(RQ) elements, where R, C, and Q represent the resistances, capacitance, and capacitive constant phase element, respectively. R_E_, R_CT_, and C_DL_ represent the electrolyte resistance, charge transfer resistance, and double-layer capacitance, respectively. The C′ increased with increasing PPy content in the composites. The relaxation frequency, given by the frequency of the C″ maximum, was relatively high; however, it decreased with increasing PPy content in the composites. The high capacitance retention observed in the CV and GCD data correlated with the relatively high EIS relaxation frequencies.

**Figure 5:**
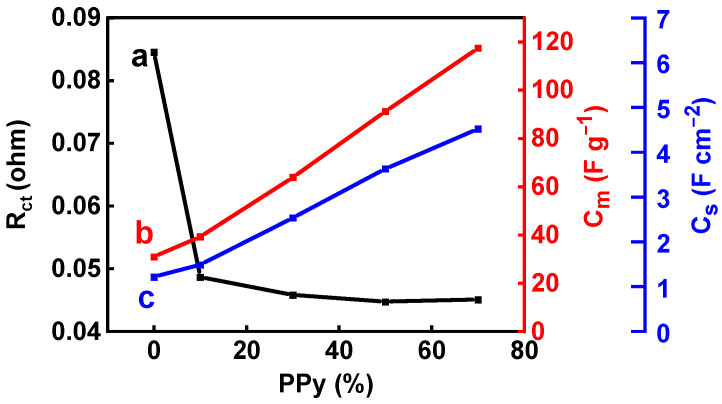
(a) R_CT_, (b) C_m_, and (c) C_S_ versus PPy content in the CuFe_2_O_4_ − PPy mixture.

**Figure 6:**
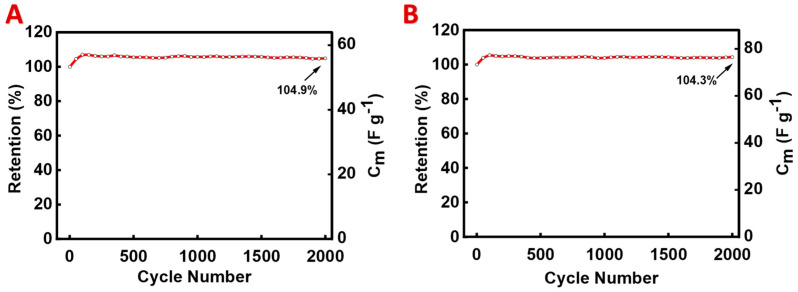
Cyclic stability data at a sweep rate of 50 mV s^−1^ for (**A**) CFO50 and (**B**) CFO30.

The analysis of the EIS data showed a significant reduction in the charge transfer resistance with the increasing PPy content in the range of 0–20% (Figure 5). As a result, at higher PPy contents, a significant capacitance increase was observed with increasing PPy concentration (Figure 5). This can result from the enhanced charge transfer at the interface of CuFe_2_O_4_ and PPy and the high capacitance of PPy. Moreover, PPy created an additional conductive percolating network in the composites. One of the problems limiting PPy applications in supercapacitor devices containing neutral electrolytes is poor cyclic stability [58]. A significant reduction in capacitance was reported during the first 1000 cycles in other composites containing magnetic particles and PPy [24]. This resulted from the poor cyclic stability of PPy. However, the investigation of the composites with high PPy contents revealed their good cyclic stability. Figure 6 shows the cycling stability test results for CFO50 and CFO30. The capacitance increased slightly at the beginning of cycling and remained nearly constant. The capacitance retention was 104.9% and 104.3% for CFO50 and CFO30, respectively, after 2000 cycles. The slight initial increase in the capacitance could be attributed to the microstructural changes within the electrode bulk.

**Figure 7:**
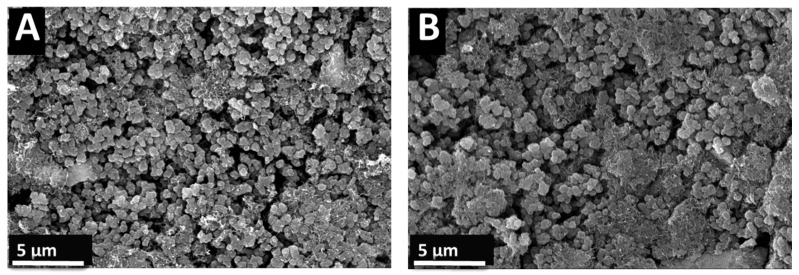
SEM images of surface microstructures of (**A**) CFO50 and (**B**) CFO30.

Figure 7 presents SEM images of the surface microstructures of the CFO50 and CFO30 composite electrodes, which showed the highest capacitances. The electrodes showed porous microstructures, which was beneficial for the electrolyte’s diffusion. The typical pore size was <1 µm. The electrode porosity resulted from the packing of CuFe_2_O_4_, PPy, and MWCNT.

**Figure 8:**
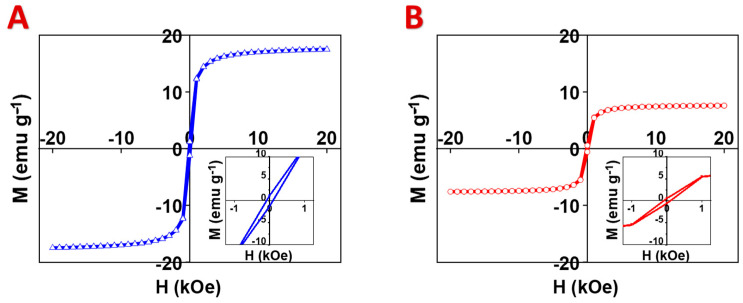
M–H curves for (**A**) CFO50 and (**B**) CFO30 (insets show low-field range).

The relatively high capacitance of CFO50 and CFO30 is promising for energy storage in supercapacitors. Such composites showed higher capacitances compared with the composites containing other spinel and hexagonal ferrite ferrimagnetic ceramics with the same mass loading in the same potential range [24,25]. Moreover, CFO50 and CFO30 showed relatively high magnetization. Figure 8 shows the M–H curves plotted from the vibration magnetometer data. The CFO50 and CFO30 composites showed saturation magnetization values of 17.5 and 7.6 emu g^−1^, respectively. Those values were lower than the CFO100 magnetization of 31.5 emu g^−1^ due to the lower CuFe_2_O_4_ content in the electrode. The coercivity values (H_c_) from the hysteresis curves indicate that the CFO50 and CFO30 samples showed soft ferrimagnetic behavior, with H_c_ values between 50 and 100 Oe and a small remanent magnetization. This can result from the interaction of CuFe_2_O_4_ with the paramagnetic PPy [59,60]. The results of this study show that the properties of CuFe_2_O_4_ composites can be varied. Such composites belong to the category of magnetic pseudocapacitors [44], which are promising for various applications based on magnetoelectric and magnetocapacitive effects. The observation of capacitive properties in high-active-mass composites depends on using advanced Ni foam current collectors, which offer high corrosion resistance, conductivity, and porosity. However, the investigation of magnetocapacitive effects in electrodes prepared in this investigation presented difficulties since Ni is ferromagnetic, which generates its own magnetic field in an external magnetic field. Thus, non-magnetic current collectors with similar properties are needed. It should be noted that the composite CuFe_2_O_4_ spinel–PPy materials prepared in this investigation showed higher capacitances compared with other polymer composites containing various ferrimagnetic spinel compounds [40,61,62,63].

## 4. Conclusions

Spinel CuFe_2_O_4_ nanoparticles with a crystal size of 20–30 nm and saturation magnetization of 31.4 emu g^−1^ were prepared by hydrothermal synthesis and used for the fabrication of CuFe_2_O_4_-PPy composites. The maximum PPy content in the composite electrodes of the same mass and thickness was 70%. The addition of PPy to CuFe_2_O_4_ reduced the charge transfer resistance and improved the material utilization at a high-active-mass loading of 40 mg cm^−2^, enabling electrode fabrication with high C_S_ and C_m_ values. The capacitance increased with the increasing PPy content in the composites. The CFO50 and CFO30 composites showed the highest capacitances, which were 3.60 and 4.52 F cm^−2^ at 1 mVs^−1^ or 3.90 and 4.60 at 3 mA cm^−2^, respectively. The obtained capacitances were higher compared with the capacitances of other composites of magnetic ceramic particles and PPy. The capacitance and magnetic properties of the composites can be varied. The magnetic behavior of the composites is influenced not only by the ferrimagnetic CuFe_2_O_4_ but also by the paramagnetic PPy. The composites belong to the category of magnetic supercapacitor materials and can potentially be used for energy storage and applications based on their magnetoelectric and magnetocapacitive properties. The use of ferromagnetic Ni for the fabrication of current collectors generates difficulties in the investigation of such properties. Future research must be focused on the replacement of commercial Ni foam current collectors with non-magnetic conductive current collectors for the investigation of magnetocapacitive and magnetoelectric effects. It is also expected that the development of new composites of conductive redox-active polymers with other magnetic materials and their solid solutions can result in advanced electrodes for energy storage and the capacitive deionization of water.

## Figures and Tables

**Table 1:** Capacitances of different composite electrodes.

Electrode	Capacitance from CV Data				Capacitance from GCD Data			
	1 mV s^−1^		100 mV s^−1^		3 mA cm^−2^		40 mA cm^−2^	
	C_S_ F cm^−2^	C_m_ F g^−1^	C_S_ F cm^−2^	C_m_ F g^−1^	C_S_ F cm^−2^	C_m_ F g^−1^	C_S_ F cm^−2^	C_m_ F g^−1^
CFO 100	1.2	31	0.85	21.7	1.17	29.9	0.63	16.14
CFO 90	1.5	39.2	1.1	29.35	1.46	38.5	1.22	32
CFO 70	2.53	63.8	2.07	52.2	2.6	66.7	2.22	55.84
CFO 50	3.6	91.1	1.87	47.1	3.9	113	2.7	68
CFO 30	4.52	117.37	2.24	58.4	4.6	120.1	3.56	92.68

## Data Availability

The data are available upon request.
